# The ventilatory component of the muscle metaboreflex is overstimulated in transthyretin cardiac amyloidosis patients with poor aerobic capacity

**DOI:** 10.3389/fphys.2023.1174645

**Published:** 2023-05-15

**Authors:** Astrid Monfort, Eugenie Thevenet, Lievyn Enette, Cedric Fagour, Jocelyn Inamo, Remi Neviere

**Affiliations:** ^1^ Department of Cardiology, CHU Martinique (University Hospital of Martinique), Fort de France, France; ^2^ Cardiovascular Research Team EA7525, Université des Antilles, Fort de France, France; ^3^ Department of Endocrinology, CHU Martinique (University Hospital of Martinique), Fort de France, France; ^4^ Department of Clinical Physiology, CHU Martinique (University Hospital of Martinique), Fort de France, France

**Keywords:** transthyretin, cardiopulmonary exercise testing, autonomic dysfunction, transthyretin cardiac amyloidosis, metaboreflex

## Abstract

**Background:** The exercise pressor reflex, i.e., metabo- and mechano-reflex, partially regulates the control of ventilation and cardiovascular function during exercise. Abnormal exercise pressor reflex response has been associated with exaggerated ventilatory drive, sympathovagal imbalance and exercise limitation in chronic heart failure patients. Whether metaboreflex is over-activated and participate to poor aerobic capacity in patients with hereditary transthyretin cardiac amyloidosis (CA-TTR) is unknown.

**Methods:** Twenty-two CA-TTR patients (aged 76 ± 7, 68% male) with the V122I (p.Val142Ile) transthyretin underwent a thorough evaluation including heart rate variability metrics, electrochemical skin conductance (ESC), physical function cardiopulmonary exercise testing, and muscle metaboreflex assessment. Eleven control subjects were chosen for muscle metaboreflex assessment.

**Results:** Age-matched controls (*n* = 11) and CA-TTR patients (*n* = 22) had similar metaboreflex sensitivity for heart rate, stroke volume, cardiac index and mean systemic arterial pressure. Compared with age-matched controls, metaboreflex sensitivity for systemic vascular resistance (−18.64% ± 6.91% vs 3.14% ± 23.35%) and minute-ventilation responses (−9.65% ± 14.83% vs 11.84% ± 23.1%) was markedly increased in CA-TTR patients. Values of ESC displayed positive correlations with stroke volume (*r* = 0.53, *p* = 0.011) and cardiac index (*r* = 0.51, *p* = 0.015) components of metaboreflex sensitivity, an inverse correlation with systemic vascular resistance (*r* = −0.55, *p* = 0.008) and a trend with mean arterial (*r* = −0.42, *p* = 0.052) components of metaboreflex sensitivity. Peak aerobic capacity (peak VO_2_%) displayed an inverse correlation with the ventilation component of metaboreflex sensitivity (*r* = −0.62, *p* = 0.015).

**Conclusion:** Consistent with the “muscle hypothesis” in heart failure, it is proposed that deterioration of skeletal muscle function in hereditary CA-TTR patients may activate muscle metaboreflex, leading to an increase in ventilation and sensation of breathlessness, the perception of fatigue, and overall sympathetic activation.

## 1 Introduction

Hereditary transthyretin mediated amyloidosis, i.e., ATTRv, is a rare, autosomal dominant, fatal disease, in which systemic amyloid progressively damages multiple organs leading to disability and death (Crisafulli et al., 2007; Ichinose et al., 2012; Kharoubi et al., 2021; Maurer et al., 2026). The most common transthyretin mutation in African Americans in the United States. and African descent in the Caribbean is V122I (p.Val142Ile) (Dungu et al., 2016; Batra et al., 2020; Monfort et al., 2020). Patients carrying p. Val142Ile mutation typically develop a progressive restrictive cardiomyopathy (CA-TTR) caused by extracellular deposition of abnormal transthyretin in the myocardium and less frequently features of peripheral neuropathy (Dungu et al., 2016; Clemmensen et al., 2017; Maurer et al., 2019; Batra et al., 2020; Kittleson et al., 2020; Kaku et al., 2022; Smiley et al., 2022; Maurer et al., 2026). Neurological manifestations in CA-TTR patients include sensorimotor and autonomic neuropathy, which predominantly involves small non-myelinated nerve fibers (Algalarrondo et al., 2106; Goldstein, 2016; Griffin et al., 2021; Kaur et al., 2021; Kharoubi et al., 2021; Tozza et al., 2021). Sensory loss, mainly affecting pain and temperature sensation, is followed a progressive involvement of motor fibers leading to leg weakness and gait abnormalities. Autonomic neuropathy may also involve the heart, leading to orthostatic hypotension, reduced heart rate variability, arrhythmias, heart block, as well as cardiac parasympathetic cholinergic and sympathetic noradrenergic denervation (Algalarrondo et al., 2106; Goldstein, 2016; Monfort et al., 2020).

Beside direct myocardial dysfunction, autonomic imbalance and abnormal reflex regulation of the cardio-pulmonary system, i.e., muscle ergoreflex activity, contribute to muscle fatigue and breathlessness leading to exercise limitation. The ergoreflex, that combines metabo- and mechano-reflexes, is a cardiorespiratory reflex activated by exercise coupling ventilation and cardiovascular function to exercise intensity (Alam and Smirk, 1937; Aimo et al., 2021; Gama et al., 2021). While the mechano-reflex is activated by mechanical stretching, the metaboreflex is activated by accumulation of metabolites in the exercising muscle humans, which are sensed by receptors in the muscle interstitial space and transmitted to the central nervous system via small non-myelinated group IV C fibers (Crisafulli, 2017; Aimo et al., 2021; Gama et al., 2021; Teixeira and Vianna, 2022). In response to metaboreflex stimulation, several regions of the central nervous system are activated, ensuring an adequate cardiorespiratory response during exercise mainly through increased sympathetic outflow. Metaboreflex can be clinically explored using post-exercise circulatory occlusion by the inflation of a cuff to a supra-systolic pressure immediately prior to the end of exercise and throughout recovery thereby trapping exercise-induced metabolites. Metaboreflex activation induced by post exercise muscle ischemia (PEMI) has been explored in normal subjects and pathological conditions such as chronic heart failure (Aimo et al., 2021; Barrett-O'Keefe et al., 2018; Crisafulli, 2017; Crisafulli et al., 2007; Ichinose et al., 2012; Kaur et al., 2018a; Kaur et al., 2018b; Piepoli and Crisafulli, 2014; Piepoli et al., 2006) and in patients with primary muscle disorders (Aimo et al., 2021; Gama et al., 2021; Giannoni et al., 2017). Systematic reviews have concluded that the metaboreflex is exaggerated in chronic heart failure patients driving peripheral vasoconstriction and exercise limitation (Crisafulli et al., 2007; Piepoli and Crisafulli, 2014; Crisafulli, 2017; Aimo et al., 2021; Gama et al., 2021).

Whether metaboreflex is over-activated and may participate to poor aerobic capacity in CA-TTR patients is unknown. In the present study, we aimed to determine whether metaboreflex is over activated in CA-TTR patients, as well as to assess its relationships with autonomic imbalance and impaired aerobic capacity.

## 2 Patients and methods

### 2.1 Patients

Participants were recruited at the Department of Cardiology of the University Hospital of Martinique from September 2019 to May 2021. Consecutive patients with the diagnosis of hereditary V122I (p.Val142Ile) transthyretin cardiac amyloidosis (CA-TTR) were prospectively enrolled. All patients declared African ancestries. Information regarding the study protocol and main objectives were provided to all patients. Risks and description of the different procedures were explained to the patients. All patients were managed in accordance with the amended Declaration of Helsinki (http://www.wma.net/en/30publications/10policies/b3/). Informed written consent was given prior to the inclusion of patients in the study. The study was approved by the CHU Martinique hospital’s institutional review board (IRB #01022019). Subjects having coronary artery disease or/and chronic obstructive pulmonary disease were excluded. Subjects were also excluded if they were taking tobacco products or more than 10 alcoholic beverages per week. Only patients with NYHA functional class II or higher status were enrolled. Assessment of cardiac function, cardiopulmonary exercise testing and metaboreflex sensitivity were performed during a maximum time interval of 4 weeks. Eleven healthy controls were matched to patients with respect to age and sex, and underwent metaboreflex assessment and cardiopulmonary exercise testing. Informed written consent was given prior to the inclusion of healthy control subjects in the study.

### 2.2 Diagnosis of amyloidosis

Transthyretin amyloidosis was diagnosed by cardiac uptake on ^99m^Tc-labeled bone tracer scintigraphy and typical abnormalities on cardiac magnetic resonance imaging or echocardiography in the absence of monoclonal gammopathy or abnormal free light chains in blood and urine (Kittleson et al., 2020). Abnormalities on cardiac magnetic resonance imaging suggesting CA-TTR included T1 and extracellular volume mapping lengthening and abnormal late gadolinium enhancement in a non-coronary distribution. Nuclear imaging (General Electric Medical Systems SPECT gamma camera Discovery) displayed cardiac uptake grade ≥2 of the Perugini classification of bone tracer technetium^99m^labeled hydroxy methylene diphosphonate (^99m^Tc-HMDP). Cardiac echography (EPIQ 7C Philips Healthcare, Suresnes, France) was performed according to standard technique following the American Society of Echocardiography guidelines. Abnormalities on echography suggesting CA-TTR included left ventricular hypertrophy and abnormal myocardial texture characterized as a speckled appearance. Abnormal myocardial texture was defined by granular sparkling of the myocardial walls on echocardiography. Increased wall thickness score (IWT score), which incorporates relative wall thickness (RWT), E/e′, tricuspid annular plane systolic excursion (TAPSE), longitudinal strain and systolic apex-to-base (SAB) ratio was calculated (Boldrini et al., 2020). A IWT score above 8 is indicative of CA with a sensitivity of 46% and a specificity of 98% (Boldrini et al., 2020). Cardiac biomarker evaluation included plasma NT-proBNP and high‐sensitivity troponin levels. The National Amyloidosis Centre (NAC) transthyretin amyloidosis (ATTR) stage was calculated for each patient (Gillmore et al., 2017). Genetic testing for TTR mutation was performed in all TTR amyloidosis.

Medical history or signs of neurological involvement was also investigated by a senior specialist in amyloidosis. Signs or medical history of diffuse neuropathy (including pain, numbness, weakness, paresthesia and urinary bladder disturbances), carpal tunnel syndrome, spinal stenosis were sought for all patients. The presence of dysautonomia (including orthostatic hypotension, nausea and vomiting, diarrhea, constipation, fecal incontinence, disturbances in bladder function) was recorded.

### 2.3 Electrochemical skin conductance

Sudomotor function was assessed via Sudoscan^®^ (Impeto Medical, Paris, France). Patients, in standing position, were asked to place their hands and feet on the devices’ electrodes for 3 min. An electrical stimulus (<4 V) was automatically generated. The electrochemical skin conductance (ESC), measured in micro-Siemens (μS), was recorded. Sudoscan^®^ test results are provided as hands (hESC) and feet (fESC) conductance (mean of left and right side). Sudomotor function was considered normal with values of hESC above 60 μS and/or fESC above 70 μS (Mayaudon et al., 2010; Casellini et al., 2013; Hussein et al., 2021; Kharoubi et al., 2021).

### 2.4 Heart rate variability metrics

Heart variability metrics were extracted from the patients’file as part of routine care, which included 24-h Holter monitoring using a 3-channel—7 electrodes device (ECG Spider View) with a sampling rate recording of 200 Hz. Heart rate variability was evaluated in patients with sinusal rhythm. The system was programmed to automatically capture all ectopic beats or rhythmic disturbances. Algorithm-based methods filtered for and excluded all non-sinus beats. Validity of exclusion was confirmed by the operator. The root mean square of successive differences between normal heartbeats (RMSSD) was obtained over a 5 min recording period by first calculating each successive time difference between heartbeats in msec. Each of the values was then squared and the result was averaged before the square root of the total is obtained. The standard deviation of adjacent NN intervals (SDNN), the number of adjacent NN intervals that differ from each other by more than 50 ms (NN50) and its percentage (pNN50) were obtained during a 5 min epoch. (Abhishekh et al., 2013; Shaffer and Ginsberg, 2017).

### 2.5 Short physical performance battery

Physical function was assessed by the Short Physical Performance Battery (SPPB), developed by the National Institute on Aging (NIA) (Guralnik et al., 1994). The SPPB is composed of three different tests: a) static balance, consisting of positioning the feet in three different positions; b) chair stand test (five times sit-to-stand), consisting of getting up completely from the chair as quickly as possible five times in a row, without stopping between repetitions; and c) gait speed test, consisting of walking a distance of 4 m twice, with the fastest time being used to calculate the result (m/s). SPPB scores of 0–6, 7–9, and 10–12 indicated poor, moderate, and good physical function, respectively.

### 2.6 Cardiopulmonary exercise test

Subjects underwent a cardiopulmonary exercise test (CPET) on an upright electromagnetically braked cycle ergometer. The test exercise protocol involved an initial 3-min rest period, followed by unloaded cycling for 2 min with a progressive 5 to 10W-increment every minute until exhaustion at a pedaling frequency of 60-65 revolutions/minute (rpm), so as to reach a maximum power output within 8–12 min. The Borg scale was used for the assessment of perceived exertion and dyspnea. Minute ventilation (V_E_), oxygen uptake (VO_2_) and pulmonary CO_2_ output (VCO_2_) were measured through breath-to-breath gas analysis (PowerCube-Ergo, Ganshorn Medizin Electronic GmbH, Niederlauer, Germany). Peak VO_2_ was defined as the highest value of VO2 attained upon an incremental exercise test designed to bring the subject to the limit of tolerance. Peak VO_2_ was computed as the highest 30-s average of oxygen consumption and was normalized by the predicted VO_2_ to derive a percent predicted value according to Wasserman equation (American Thoracic Society, American College of Chest Physicians and American College of Chest Physicians, 2003). Ventilatory reserve RV at peak exercise was calculated as $RV=\frac{\left[MVV-peak\;VE\right]}{MVV}\times 100$ , where MVV is maximal voluntary ventilation estimated as FEV_1_ multiplied by 35. Peak oxygen pulse (O_2_ pulse) was calculated and was expressed in mL per beat and as percentage of predicted value by dividing the predicted peak VO_2_ by predicted peak HR (American Thoracic Society, American College of Chest Physicians and American College of Chest Physicians, 2003). Ventilatory efficiency, as indicated by the increment in V_E_ relative to VCO_2_ (V_E_/VCO_2_ slope) was calculated off-line as a linear regression function using 10-s averaged values and excluding the non-linear part of the relationship after the respiratory compensation point (where non-linear rise in V_E_ occurred relative to VCO_2_ in the presence of decrease of end-tidal pressure of CO_2_) (American Thoracic Society, American College of Chest Physicians and American College of Chest Physicians, 2003). Exercise was considered maximal if two of the following occurred: predicted maximal work is achieved, predicted maximal heart rate is achieved, VE/VO2 > 45 and RER >1.15, as described in the American Thoracic Society (ATS) and the American College of Chest Physicians (ACCP) statements (American Thoracic Society, American College of Chest Physicians and American College of Chest Physicians, 2003).

### 2.7 Handgrip and post exercise muscle ischemia procedures

**FIGURE 1:**
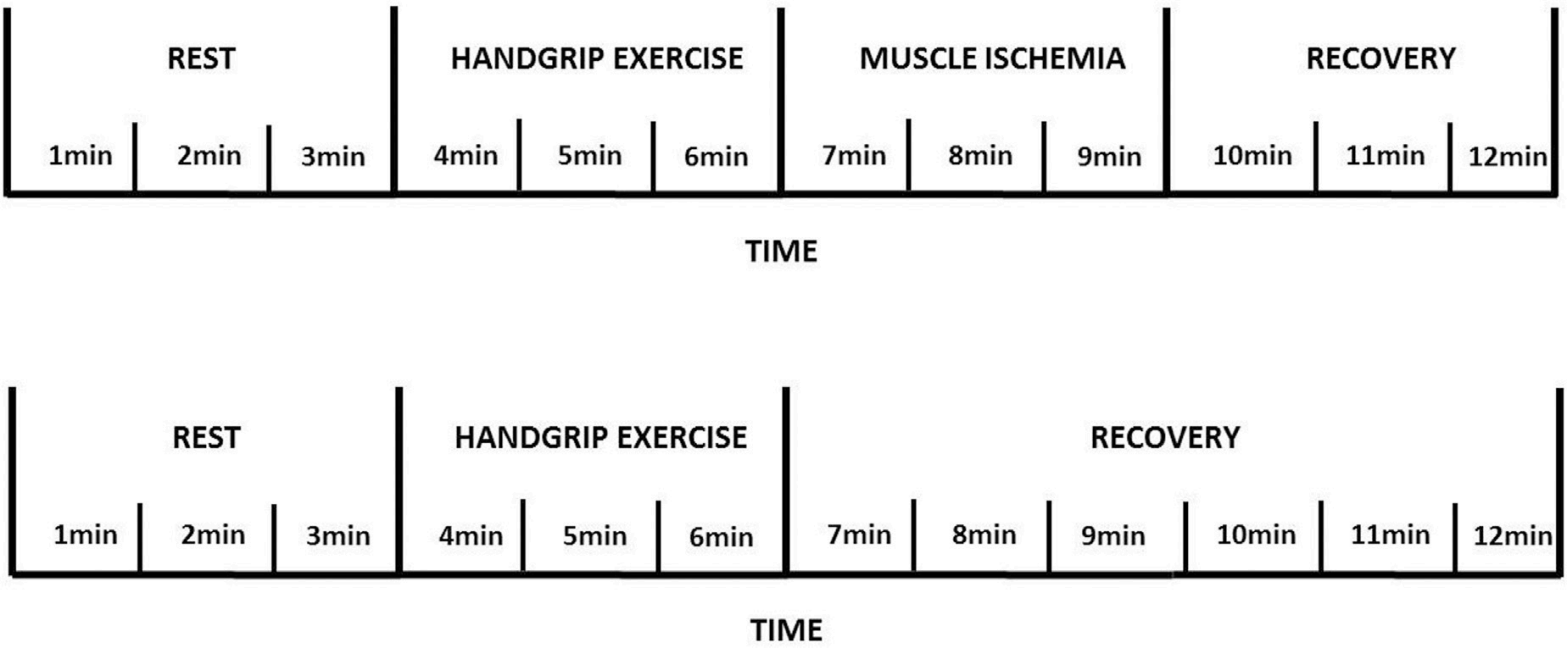
Metaboreflex assessment using handgrip exercise and post exercise muscle ischemia procedure.

Participants were instructed to fast for 2 h, to avoid physical exercises in the 48 h, and caffeine or alcohol in the 12 h prior to experimental sessions. Subjects were positioned in a seated position for 30 min with a handgrip dynamometer held in the dominant hand with the limb supported before performing handgrip strength test. Handgrip protocol used in our study (Figure 1) has been previously described (Aimo et al., 2021; Barrett-O'Keefe et al., 2018; Crisafulli, 2017; Crisafulli et al., 2007; Piepoli et al., 2006). Maximal voluntary contraction (average of five trials) of the dominant arm was first obtained by the use of a handgrip dynamometer (CAMRY Digital Hand Dynamometer, South El Monte, CA, United States). Individuals then underwent a 3 min rest period, followed by 3 min of exercise, which consisted of rhythmic (30 compressions/min) dynamic handgrip at 30% of the maximum voluntary contraction. A 3-min period of post exercise muscle ischemia (PEMI) on the exercised arm was obtained by rapidly (in less than 3 s) inflating a tourniquet to 30 mmHg above peak systolic pressure observed during exercise. The duration of circulatory arrest was 3 min. At the end of PEMI, the cuff was deflated and a further period of 3 min of recovery was allowed. This protocol has been shown to trap the muscle metabolites in the exercising limb and to maintain stimulation of the metaboreceptors. The control exercise recovery (CER) session, consisting of the same rest-exercise protocol used for PEMI, was performed followed by a CER of 6 min without tourniquet inflation. PEMI and CER sessions were spaced by a 30-min interval during which the study subject rested on a chair to completely recover.

All experiments were carried out in a temperature-controlled, air-conditioned room (temperature set at 24°C and relative humidity 60%). Hemodynamic parameters were measured throughout all phases of the study. Blood pressure (BP) was monitored noninvasively from an automatic blood pressure cuff (Tango M2 Stress Test Monitor, SunTech Medical, Inc., Morrisville, NC, United States), which allows to measure BP within less than a minute thank to a rapid deflate rate setup (>8 mm Hg/s) (White et al., 1989). Cardiac output (CO) was estimated using thoracic bioimpedance that used changes in transthoracic impedance during cardiac ejection to calculate stroke volume (PhysioFlow^®^, Manatec Biomedical, Paris, France). Physioflow^®^ device has been previously validated for the non-invasive evaluation of cardiac output at rest and during exercise (Moore et al., 1992; Charloux et al., 2000; Richard et al., 2001). For stroke volume (SV) measurements, six electrodes (Skintact FS-50) were used (2 on the neck, 2 at the xiphi-sternum and 1 on each side of the chest) after gently skin scraping. Heart rate determination was based on the R-R interval duration determined using the first derivative of the electrocardiogram (ECG). After calibration method, continuous hemodynamic measurements were performed. Adequate signal quality for interpretation was ascertain in all cases. Cardiovascular measurements were performed every minute throughout the study procedures. Systemic vascular resistance (SVR) was obtained by multiplying the mean arterial blood pressure to cardiac output ratio by 80, where 80 is a conversion factor to convert units to standard resistance units (Piepoli et al., 2006; Crisafulli et al., 2007). V_E_, VO_2_ and VCO_2_ were assessed using the same equipment described for the preliminary incremental cycle-ergometer test. All devices used during handgrip test were connected through an A/D interface that fed a Powerlab 16/35 acquisition system. Data were acquired at 1 kHz and simultaneously displayed and recorded on a desktop computer using LabChart 8 Pro (ADInstruments). Selections of filters including high, low and band-pass/stop options were setup to provide optimal data acquisition of each recorded signal.

### 2.8 Metaboreflex sensitivity definition

Ventilatory and hemodynamic components of the metaboreflex, i.e., metaboreflex sensitivity was quantified as the percentage of ventilatory minute and hemodynamic parameter (heart rate, stroke volume, cardiac index, mean arterial pressure and mean systemic vascular resistance) responses to exercise maintained by circulatory occlusion during the third minute compared with the third minute of basal recovery. As previously reported (Aimo et al., 2021), metaboreflex sensitivity was calculated as $\left[\left[\frac{Rec}{Ex}\right]\;PEMI-\left[\frac{Rec}{Ex}\right]\;CER\right]\;x\;100$ , where Ex is the mean minute-ventilation or hemodynamic parameter response during the last 30 s of exercise, Rec the mean minute-ventilation or hemodynamic parameter response during the last 30 s of the third minute of recovery, and all measures are performed during the circulatory occlusion bout (PEMI) and the “no circulatory occlusion” bout (CER) (Aimo et al., 2021).

### 2.9 Statistical analysis

We conducted a pilot and exploratory study along with the purpose of testing the hypothesis of interest. Sample size calculation was based on heart failure differences and exercise pressor reflex studies (Barrett-O'Keefe et al., 2018; Crisafulli, 2017; Guralnik et al., 1994; Nativi-Nicolau et al., 2019). Assuming alpha = 0.05 and power = 85%, a minimal sample size of 20 subjects was planned in order to highlight differences between groups and conditions. The Shapiro-Wilk test was applied to determine if the estimated parameters are well-modeled by a normal distribution. Continuous variables were summarized using either mean ± standard deviation (SD) or median and inter-quartile range [25-75^th^], while categorical variables were listed as frequencies and percentages. Hemodynamic and breath-by-breath ventilatory collected data were averaged for the last 30 s of the corresponding condition. Independent samples *t*-test were performed for comparisons between groups of continuous variables if normally distributed, while skewed data were tested with the Kruskal–Wallis test. Differences between groups of absolute values of variables during rest periods preceding handgrip runs were studied by means of the two-way analysis of variance (ANOVA) for repeated measures with group (CA-TTR or control patients) and condition (rest before CER or before PEMI test) as main factors followed by Tukey’s *post hoc* test when appropriate. Statistical analyses were performed using the Statistical Package for the Social Sciences (SPSS) version 18.0 for Windows (SPSS, Inc., Chicago, IL). The level of statistical significance was set at *p*-value <0.05.

## 3 Results

### 3.1 Characteristics of control subjects and CA-TTR patients

Eleven control subjects (*n* = 11) and twenty two CA-TTR patients (*n* = 22) with the transthyretin V122I (p.Val142Ile) mutation were enrolled. Main characteristics of control subjects and CA-TTR patients are displayed Table 1. ECG, echocardiographic parameters, cardiac biomarkers, functional capacity and electrochemical skin conductance were collected during the year following the diagnose of cardiac amyloidosis and are presented Table 1. We found that display peripheral neuropathy and dysautonomia identified on neurological examination was not uncommon in CA-TTR patients carrying the transthyretin V122I (p.Val142Ile) mutation (Table 1). In this population, 33% of patients had symptoms compatible with peripheral neuropathy (mostly pain, numbness, and weakness). Dysautonomia was suggested by results of electrochemical skin conductance (ESC) and heart rate variability metrics (Table 1). In CA-TRR patients, fESC and hESC were under normal values (<70 μS and 60 μS, respectively) in 59% and 64% of cases. Physical function evaluated by SPPB scoring and maximal aerobic capacity were markedly reduced in CA-TTR patients (Table 1). All patients were under conventional treatment regimen for heart failure and cardiac amyloidosis. All but one patient had tafamidis, a stabilizer of the native tetramer structure of TTR, which was clinically indicated in this series of patients with genetic testing-proven hereditary TTR-cardiomyopathy.

**TABLE 1:** Characteristics of patients with transthyretin cardiac amyloidosis (CA-TTR).

	CA-TTR patients *n* = 22	Controls *n* = 11
Age at diagnostic, year	76 ± 7	73 ± 3
Sex (male/female), n (%)	15 (68)/7 (32)	7 (64)/4 (36)
Body mass index (BMI), kg.m^-2^	26 ± 8	27 ± 3
NYHA Class I/II/III, n (%)	15)/15(68)/6(27)	9(82)/2(18)/00)
Resting systolic blood pressure, mmHg	120 ± 22	134 ± 12
Resting diastolic blood pressure, mmHg	74 ± 10	82 ± 11
**Clinical history**		
Transthyretin mutation p.Ile122Val, n (%)	22 (100)	N/A
Systemic hypertension, n (%)	13 (59)	0 0)
Diabetes, n (%)	5 (23)	0 0)
Sleep apnea syndrome, *n* (%)^n=18^	1 6)	0 0)
Carpal tunnel syndrome, *n* (%)^n=20^	13 (65)	0 0)
Peripheral neuropathy, *n* (%)^n=21^	7 (33)	0 0)
History of atrial fibrillation, n (%)	10 (45)	0 0)
Pacemaker, n (%)	0 0)	0 0)
Implantable cardioverter–defibrillator, n (%)	0 0)	0 0)
Dupuytren’s syndrome, n (%)	2 9)	0 0)
Gastrointestinal dysautonomia, n (%)^n=20^	5 (25)	0 0)
**Biomarkers**		
NT- proBNP, pg.ml^-1^ (median [IQR])	1510 [670-2044]	N/A
High-sensitivity Troponin, ng. L^-1^ (median [IQR])	100 [42-165]	N/A
Creatinine, μmol.L^-1^ (median [IQR])	110 [83-134]	N/A
eGFR, mL.min^-1^.1.73 m^-2^ (median [IQR])	70 [52-98]	N/A
ATTR Biomarker staging I,II,III, n (%)	5 (23),7 (32), 10 (45)	N/A
**ECG**		
Sinus rhythm, n (%)	20 (91)	N/A
Pseudo-infarction pattern, n (%)	10 (45)	N/A
Low QRS voltage (<0.5 mV), n (%)	13 (59)	N/A
Resting heart rate, beat.min-1	83 ± 12	76 ± 7
**24-h ECG Holter**		
- Recording duration, h	22.3 ± 0.8	N/A
- SDNN, msec (median [IQR])	95 [78-130]	N/A
- NN50, msec (median [IQR])	104 [81-133]	N/A
- pNN50, % (median [IQR])	16 [4-46]	N/A
- RMSSD, msec (median [IQR])	61 [27-105]	N/A
**Scintigraphy([99mTc]-HMDP**		
Perugini score 2/3, n (%)	5 (22)/18 (78)	N/A
**Echocardiography**		
Interventricular septum thickness, mm (median [IQR])	15.0 [13.0-16.5]	N/A
Posterior wall thickness, mm (median [IQR])	14.0 [13.0-16.0]	N/A
LV mass, g.m^-2^ (median [IQR])	138 [106-157]	N/A
LV ejection fraction, % (median [IQR])	52 [43-60]	N/A
Cardiac index, L.min^-1^.m^-2^ (median [IQR])	2.2 [1.7-2.4]	N/A
LA volume, ml.m^-2^ (median [IQR])	42 [35-53]	N/A
E/A ratio (median [IQR])	1.6 [1.0-2.4]	N/A
E/e’ratio (median [IQR])	15.2 [11.3-17.8]	N/A
Tricuspid regurgitation velocity; m.s^-1^ (median [IQR])	2.6 [2.3-2.9]	N/A
Pulmonary artery systolic pressure, mmHg (median [IQR])	31 [26-40]	N/A
Right ventricular fractional area change, % (median [IQR])	44 [38-50]	N/A
Tricuspid annular plane systolic excursion, mm (median [IQR])	19 [15-21]	N/A
IWT score, (median [IQR])	7.00 [6.75-10.00]	
**Functional evaluation and exercise capacity**		
Short Physical Performance Battery, SPPB (points)	11 [10-11]	N/A
- Balance (score)	10 [10-10]	N/A
- Gait speed, m/sec	4 [3-5]	N/A
- Chair stand, sec	12 [11-17]	N/A
Six Minute Walk Test, m	327 [205-388]	N/A
Peak workload (upright bicycle), watts^n=20^	64 ± 18	N/A
Peak VO_2_, mL.kg^-1^.min^-1n=20^	13.0 ± 2.8	N/A
Peak VO_2_, predicted %^n=20^	58 ± 14	N/A
Peak O_2_ pulse, %^n=20^	59 ± 12	N/A
Peak RER^n=20^	1.15 ± 0.08	N/A
VE VCO_2_ slope^n=20^	41 ± 6	N/A
Peak heart rate, % maximal heart rate^n=20^	88 ± 12	N/A
Heart rate reserve used, %^n=20^	67 ± 24	N/A
Heart rate recovery (1 min), %^n=20^	7 ± 5	N/A
Heart rate recovery (3 min), %^n=20^	18 ± 10	N/A
Borg scale (leg) (median [IQR])^n=20^	5 [3.0-8.0]	N/A
Borg scale (dyspnea) (median [IQR])^n=20^	7 [3.5-8.0]	N/A
**Electrochemical skin conductance** (Sudoscan)		
Feet electrochemical skin conductance (fESC), µS	63.0 ± 13.4	N/A
Hands electrochemical skin conductance (hESC), µS	50.1 ± 18.1	N/A
**Medication**		
ACEi(s)/ARB(s), n (%)	8 (36)	0 0)
AA s), n (%)	4 (18)	0 0)
Beta-blocker, n (%)	0 0)	0 0)
Calcium channel blocker, n (%)	4 (18)	0 0)
Amiodarone, n (%)	4 (18)	0 0)
Furosemide, n (%)	19 (86)	0 0)
Anticoagulants, n (%)	17 (77)	0 0)
Tafamidis at baseline^a^, n (%)	22 (100)	0 0)

Normally distributed quantitative variables are presented as mean ± standard deviation. Skewed distributed quantitative variables are displayed as median and interquartile ranges [IQR, 25%–75%]. Absolute values (percentage) are displayed for categorical variables.

Abbreviations: ^99m^Tc HMDP: ^99m^ technetium - hydroxymethylene-diphosphonate; AA s): aldosterone antagonist(s); ACEi(s): angiotensin-converting enzyme inhibitor(s); ARB(s): angiotensin II, receptor antagonist(s); Borg: modified Borg scale (0-10) for rate of perceived exertion scale at peak exercise; E/e': early diastolic transmitral velocity to early mitral annulus diastolic velocity ratio; eGFR: estimated glomerular filtration rate; ESC: electrochemical skin conductance; IWT, score: increased wall thickness score incorporing relative wall thickness (RWT), E/e’, tricuspid annular plane systolic excursion (TAPSE), longitudinal strain and systolic apex-to-base (SAB) ratio; LA: left atrial; LV: left ventricle; NT-proBNP: N-terminal pro B-type Natriuretic Peptide; NYHA:New York Heart Association (NYHA) classification; RER: respiratory exchange ratio; RV S: peak systolic tissue Doppler velocity of the tricuspid annulus; SDNN: NN50: number of adjacent NN, intervals that differ from each other by more than 50 ms; pNN50: percentage of adjacent NN, intervals that differ from each other by more than 50 ms; RMSSD: Root mean square of successive RR, interval differences; VE: minute ventilation; VO_2_: oxygen uptake; VCO_2_: pulmonary carbon dioxide output.

^a^ Started for at least 6 months before evaluation.

ATTR, Biomarker staging: Stage I: NT-proBNP<_3000 ng/L and eGFR>_45 mL/min, Stage III: NT-proBNP>3000 ng/L and eGFR<45 mL/min, Stage II: the remainder.

### 3.2 Metaboreflex evaluation

Assessment of metaboreflex was easily performed in all patients. Rate of perceived exertion was scaled “moderate intensity” in all patients. No patients complained discomfort or pain during the ischemic bout. Age-matched controls and CA-TTR patients had similar metaboreflex sensitivity for heart rate, stroke volume, cardiac index and mean systemic arterial pressure (Table 2). Compared with age-matched controls, metaboreflex sensitivity for systemic vascular resistance and minute-ventilation responses was markedly higher in CA-TTR patients (Table 2).

**TABLE 2:** Comparisons of hemodynamic and ventilatory components of the metaboreflex between patients with transthyretin cardiac amyloidosis (CA-TTR) and controls.

Metaboreflex sensitivity	Controls (*n* = 11)	CA-TTR (*n* = 22)	P
Heart rate, %	1.16 ± 6.56	−0.33 ± 7.31	0.573
Stroke volume, %	4.47 ± 8.08	1.22 ± 12.62	0.444
Cardiac index, %	4.96 ± 5.63	1.79 ± 13.60	0.466
Mean arterial pressure, %	0.16 ± 3.04	3.75 ± 18.54	0.531
Systemic vascular resistance, %	−18.64 ± 6.91	3.14 ± 23.35	0.005
Minute ventilation, %	−9.65 ± 14.83	11.84 ± 23.1	0.009

Metaboreflex sensitivity was quantified as the percentage (%) of the heart rate, stroke volume, cardiac index, mean arterial pressure, systemic vascular resistance and ventilatory response to exercise maintained by circulatory occlusion during the last 30 s of the third minute (PEMI), compared with the last 30 s of third minute of basal recovery (CER). Results are reported as mean ± SD, Mean differences among groups were evaluated through the unpaired Student’s *t*-test.

**FIGURE 2:**
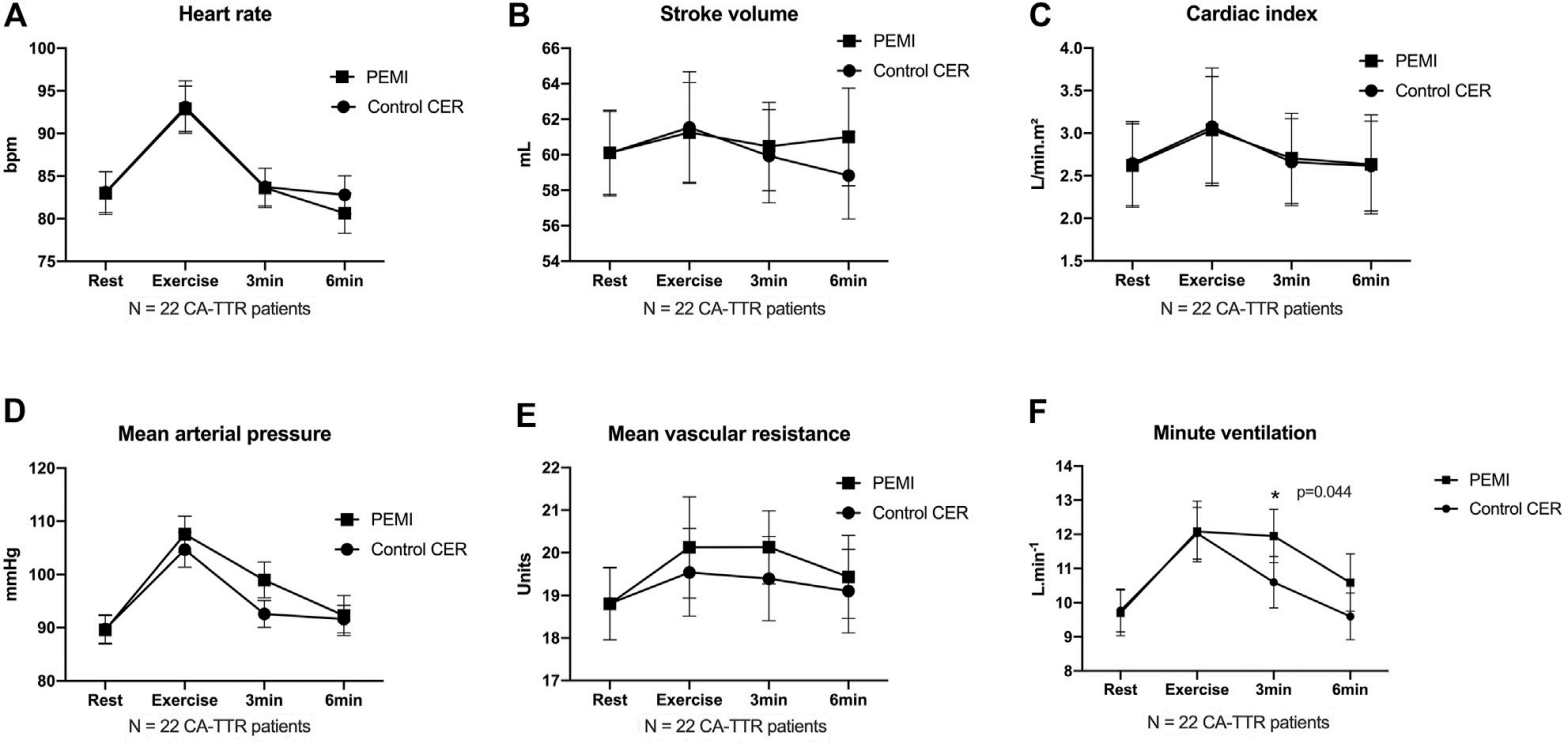
Time courses of heart rate **(A)**, stroke volume **(B)**, cardiac index **(C)**, mean arterial pressure **(D)**, mean systemic vascular resistance **(E)** and minute ventilation **(F)** at rest, during exercise, and during 3 min and 6 min of recovery in control exercise recovery (CER) and post exercise muscle ischemia (PEMI) protocol sessions in patients with transthyretin cardiac amyloidosis. Values are mean ± SE. * indicates *p* < 0.05 (two-way analysis of variance ANOVA).

Compared with CER, activation of the metaboreflex by PEMI in CA-TTR patients prolonged minute-ventilation (9.8 ± 3.0 vs 12.1 ± 3.8 L min^-1^; *p* = 0.044) at the third minute post exercise, without concomitant changes in heart rate (84 ± 10 vs 84 ± 11 beats per minute; *p* = 0.974), stroke volume (59 ± 12 vs 61 ± 12 mL m^-2^; *p* = 0.640), cardiac index (2.7 ± 0.50 vs 2.7 ± 0.5 L min^-1^. m^-2^; *p* = 0.773), mean systemic arterial pressure (93 ± 12 vs 99 ± 16 mm Hg; *p* = 0.136), and systemic vascular resistance (19 ± 5 vs 20 ± 4 units; *p* = 0.531) (Figure 2).

### 3.3 Relationships between metaboreflex sensitivity and functional parameters in CA-TTR patients

**FIGURE 3:**
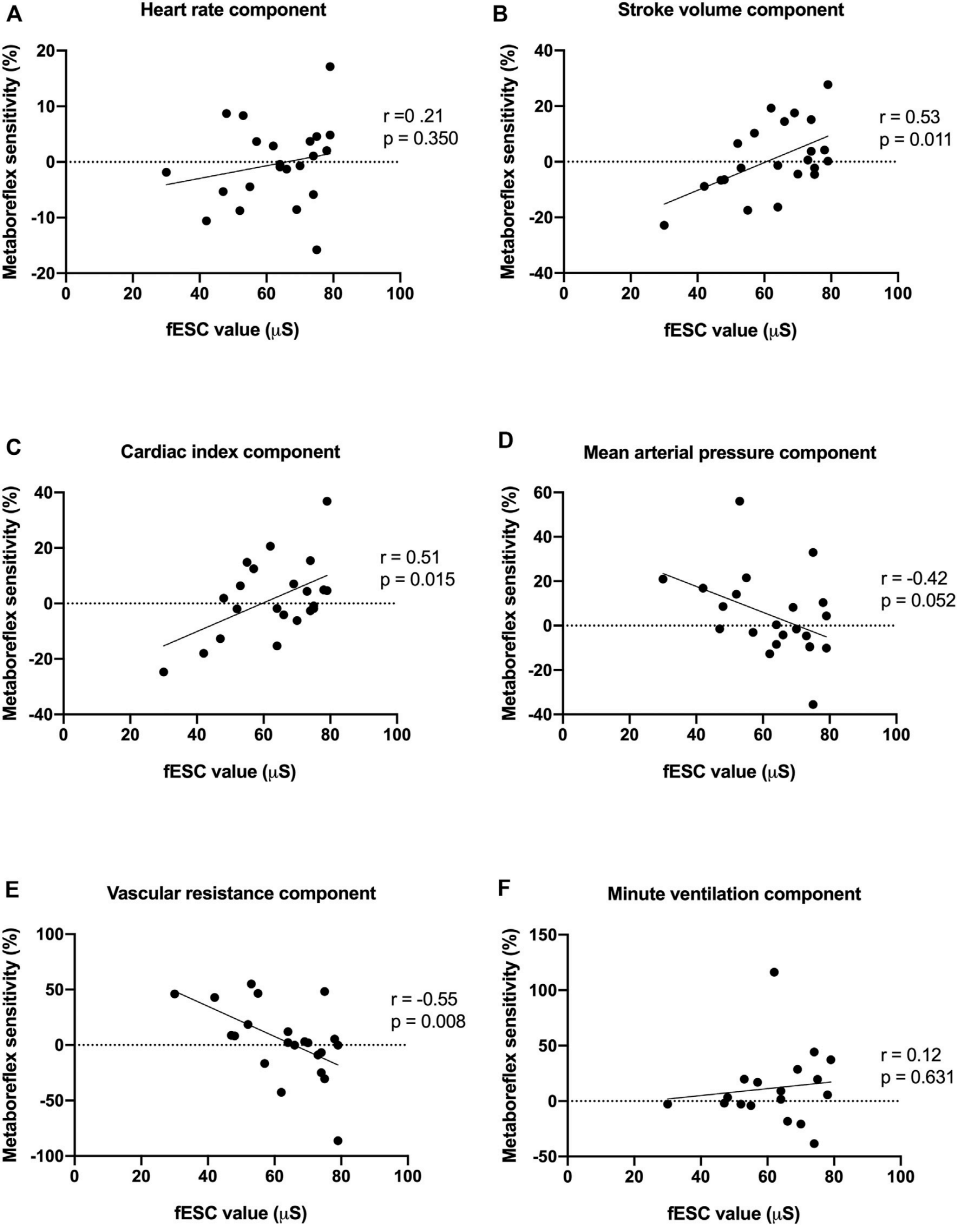
Associations between feet electrochemical skin conductance (fESC) and heart rate **(A)**, stroke volume **(B)**, cardiac index **(C)**, mean arterial pressure **(D)**, systemic vascular resistance **(E)** and minute ventilation **(F)** components of metaboreflex sensitivity. fESC displayed positive correlations with stroke volume, cardiac index components of metaboreflex sensitivity, and an inverse correlation with the systemic vascular resistance component of metaboreflex sensitivity.

**FIGURE 4:**
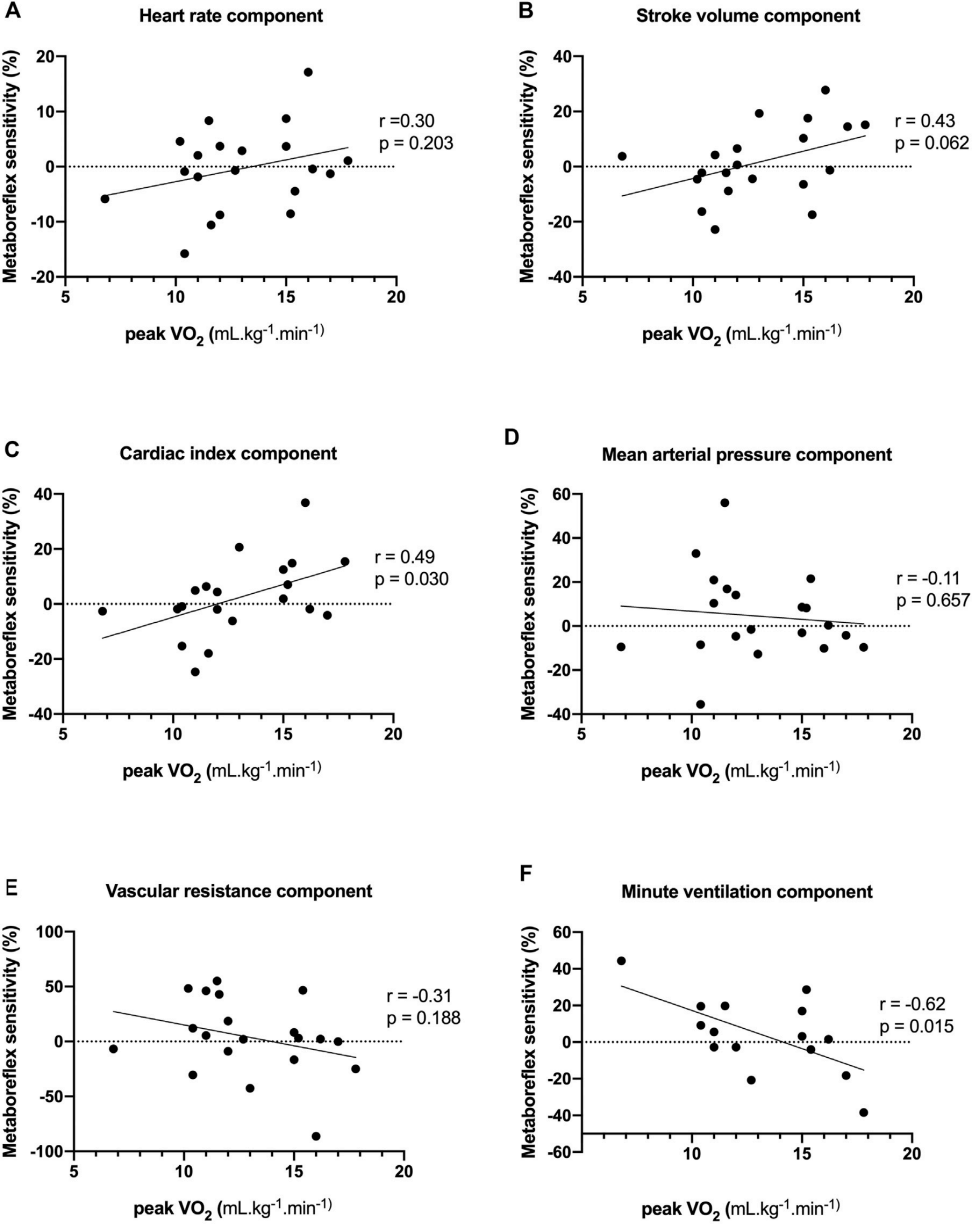
Associations between peak oxygen consumption (peak VO_2_) and heart rate **(A)**, stroke volume **(B)**, cardiac index **(C)**, mean arterial pressure **(D)**, systemic vascular resistance **(E)** and minute ventilation **(F)** components of metaboreflex sensitivity. Peak VO_2_ displayed positive correlations with cardiac index component of metaboreflex sensitivity, and an inverse correlation with the ventilation component of metaboreflex sensitivity.

Values of fESC displayed positive correlations with stroke volume and cardiac index components of metaboreflex sensitivity, an inverse correlation with systemic vascular resistance and a trend with mean arterial components of metaboreflex sensitivity (Figure 3; Table 3). Likewise, values of pNN50% displayed positive correlations with mean arterial pressure and systemic vascular resistance components of metaboreflex sensitivity (Table 3). RMSSD values displayed positive correlations with mean arterial pressure and systemic vascular resistance components of metaboreflex sensitivity, while pNN50 displayed positive correlations with mean arterial pressure and a trend with systemic vascular resistance components of metaboreflex (Table 3). Physical function scored by chair stand test and peak aerobic capacity (peak VO_2_%) displayed inverse correlations with the ventilation component of metaboreflex sensitivity, while no correlations were found with hemodynamic components of metaboreflex sensitivity (Figure 4; Table 3).

**TABLE 3:** Metaboreflex sensitivity, feet electrochemical skin conductance and performance at cardiopulmonary exercise testing in 22 patients with transthyretin cardiac amyloidosis (CA-TTR).

MS	fESC		VO_2_%		SPPB cs		V_E_VCO_2_ slope		RMSSD	
	R	P	r	P	r	P	r	P	r	P
HR	0.21	0.350	0.30	0.203	0.06	0.816	0.45	0.119	−0.16	0.786
SV	0.53	0.011	0.43	0.062	0.18	0.459	−0.12	0.700	−.0.48	0.234
CI	0.51	0.015	0.29	0.324	0.38	0.108	0.17	0.584	−0.24	0.568
MAP	−0.42	0.052	−0.11	0.657	−0.13	0.601	0.032	0.918	0.95	0.0001
SVR	−0.55	0.008	−0.31	0.188	0.18	0.458	−0.12	0.708	0.76	0.030
V_E_	0.12	0.631	−0.62	0.015	0.72	0.001	0.39	0.262	0.63	0.253

Metaboreflex sensitivity (MS) was quantified as the percentage of the heart rate (HR), stroke volume (SV), cardiac index (CI), mean arterial pressure (MAP), systemic vascular resistance (SVR) and ventilatory (V_E_) response to exercise maintained by circulatory occlusion during the third minute (PEMI), compared with the third minute of basal recovery (CER). Pearson correlation coefficient r) was used to study the possible correlation between metaboreflex sensitivity, feet electrochemical skin conductance (fESC), peak oxygen consumption (peak VO_2_% predicted), V_E_VCO_2_ slope, Short Physical Performance Battery score (SPPB, total) score, SPPB, chair stand (SPPB, cs) score and root mean square of successive RR, interval differences (RMSSD).

## 4 Discussion

This study evaluated the clinical value of metaboreflex response in Afro-Caribbean patients with hereditary cardiac amyloidosis carrying the V122I (p.Val142Ile) mutation. While this mutation is most commonly associated with cardiomyopathy (Ichinose et al., 2012; Dungu et al., 2016; Clemmensen et al., 2017; Maurer et al., 2019; Batra et al., 2020; Kittleson et al., 2020; Smiley et al., 2022; Maurer et al., 2026), growing evidence suggest that these patients may also display peripheral neuropathy and dysautonomia (Algalarrondo et al., 2106; Goldstein, 2016; Griffin et al., 2021; Kaur et al., 2021; Kharoubi et al., 2021; Tozza et al., 2021). In line, our study suggests that peripheral neuropathy and dysautonomia identified was not uncommon in this specific V122I (p.Val142Ile) mutation CA-TTR population. A hallmark of clinical features in hereditary CA-TTR patients is exercise intolerance. Of note, exercise intolerance and breathlessness have been consistently attributed to an abnormal muscle metaboreflex response in patients with chronic heart failure (Piepoli and Crisafulli, 2014; Aimo et al., 2021). Indeed, mechanoreflex and metaboreflex components of the exercise pressor reflex participate to the normal cardiovascular and ventilatory responses during physical activity by increasing sympathetic nerve activity (Alam and Smirk, 1937; Piepoli and Crisafulli, 2014; Aimo et al., 2021; Gama et al., 2021). No study so far has explored the clinical value of metaboreflex in CA-TTR patients. Herein, we report that metaboreflex sensitivity for ventilation responses was higher in CA-TTR patients than in control subjects.

Our results shed new light on the clinical significance of altered metaboreflex sensitivity and its relationship with exercise intolerance in CA-TTR patients. Age-matched controls and CA-TTR patients had similar metaboreflex sensitivity for heart rate, stroke volume, cardiac index and mean systemic arterial pressure, while mean systemic vascular resistance was increased in CA-TTR patients. Increased systemic vascular resistance component of the metaboreflex is difficult to reconcile in the absence of correlation with hemodynamic changes but may be attributed to large dispersion of blood pressure data. Autonomic dysfunction as evidenced by abnormal electrochemical skin conductance (ESC) and short-term heart rate variability metrics (RMSSD, pNN50) was associated with changes in hemodynamic components of metaboreflex sensitivity, suggesting a functional shift from a flow-mediated (i.e., cardiac output increase) to a vasoconstriction-mediated hemodynamic mechanism to achieve the target mean systemic arterial pressure during metaboreflex. Overall, these hemodynamic features of metaboreflex sensitivity in patients with CA-TTR resemble abnormalities displayed by patients with chronic heart failure (Aimo et al., 2021; Barrett-O'Keefe et al., 2018; Crisafulli, 2017; Crisafulli et al., 2007; Ichinose et al., 2012; Kaur et al., 2018a; Kaur et al., 2018b; Piepoli and Crisafulli, 2014; Piepoli et al., 2006). Correlations between low electrochemical skin conductance (ESC) and enhanced hemodynamic metaboreflex sensitivity may be representative of a generalized sympathetic dysfunction, establishing a causal link between peripheral autonomic neuropathy and metaboreflex activation. In our study, a generalized modification of group IV (small, non-myelinated) C fiber that are afferent stimuli of the exercise pressor reflex is suggested by the result of electrochemical skin conductance study in CA-TTR patients. Sudoscan^®^ is a non-invasive technology that assesses function of C-small sympathetic nerve fibers of the sweat gland via determination of chloride conductance across the skin (Mayaudon et al., 2010; Casellini et al., 2013; Hussein et al., 2021; Kharoubi et al., 2021). Low electrochemical skin conductance values have been associated with global and heart sympathetic impairment and proven to identify patients at risk of major adverse cardiovascular event (Mayaudon et al., 2010; Casellini et al., 2013; Hussein et al., 2021; Kharoubi et al., 2021). Furthermore, sudomotor dysfunction has been associated with cardiac function deterioration and poor outcome in patients with wild-type transthyretin cardiac amyloidosis (CA-TTRwt) (Kharoubi et al., 2021). Our study confirms that two-third of CA-TTR patients display low electrochemical skin conductance suggesting frequent subclinical autonomic neuropathy. When correlating metaboreflex sensitivity with fESC, our results suggest that exaggerated metaboreflex sensitivity resulted in reduced stroke volume and cardiac output along with increased mean systemic arterial pressure and systemic vascular resistance, which have been related to chronic sympathetic over activation. A sympathovagal imbalance is further supported by previous studies showing chronotropic incompetence and blunted heart rate recovery in CA-TTR patients (Nativi-Nicolau et al., 2019; Monfort et al., 2020; Nicol et al., 2021). Overall, abnormal electrochemical skin conductance and excessive metaboreflex activation in CA-TTR patients may be representative of an enhanced overall reactivity to cardiovascular sympatho-excitatory stimulus rather than a specific change in metaboreflex regulation within the skeletal muscle. Of note, such exaggerated ergoreflex sensitivity may be influenced by severity stage of heart failure or cardiovascular amyloid deposition.

Reduced physical function and peak aerobic capacity (peak VO_2_%) were associated with increased ventilation component of metaboreflex sensitivity, while no correlations were found with hemodynamic components of metaboreflex sensitivity. Association between deterioration muscle function and increased ventilation response to metaboreflex activation observed in CA-TTR patients is consistent with the “muscle hypothesis” where a primary muscle disorder increases muscle ergoreceptor sensitivity leading to exercise intolerance and sympathetic activation. Excessive ventilatory responses elicited by metaboreflex activation inversely correlated with physical function and peak VO_2_, suggesting that metaboreflex over-activation may contribute to exercise intolerance by causing hyperventilation and early onset of dyspnea in CA-TTR patients, as previously shown in chronic heart failure (Alam and Smirk, 1937; Aimo et al., 2021; Gama et al., 2021). Hence, exercise intolerance could be related to early onset of dyspnea caused by greater ventilatory metaboreflex sensitivity. This observation is consistent with previous studies showing that over-activation of the metaboreflex contributes to exercise hyperventilation and reduces exercise tolerance in patients with chronic heart failure (Alam and Smirk, 1937; Aimo et al., 2021; Gama et al., 2021). It has been proposed that changes in skeletal muscle metabolism, structure and function produces an increased activation of metabo- and mechano-receptors leading to a feedback over-activation from type III/IV muscle afferent fibers and causing heightened exercise pressor reflex with excessive arteriolar constriction and muscle hypoperfusion (Aimo et al., 2021; Gama et al., 2021; Teixeira and Vianna, 2022). Restrained muscle perfusion may potentially contribute to the early development of peripheral muscle fatigue and exercise intolerance shown by patients with cardiac amyloidosis. In line, our results suggest that exaggerated metaboreflex may represent a major determinant of exercise intolerance in patients with CA-TTR.

## 5 Study limitations

The different components of dysautonomia, which involves enteric nervous system, parasympathetic nervous system, and sympathetic nervous system abnormalities, were not evaluated in our study. Sympathetic impairment evaluated by sudomotor dysfunction was the only neurological test performed to assess dysautonomia. Cardio-vagal parasympathetic function was not evaluated. Likewise, demonstration of cardiac dysautonomia using long-term measurements of heart rate variability, arterial blood pressure response to postural change and plasma catecholamine levels were not performed in our study. The search for the role of muscle metaboreceptors in cardiopulmonary control has been largely derived from the effects of vascular occlusion. Because ischemic contractions, i.e., occlusion during exercise, are painful, human studies on the metaboreflex have focused their attention on the recovery period. Post-exercise occlusion may however produce a significant discomfort and painful sensations in some subjects, altering the ventilatory and cardiovascular responses. In addition, post-exercise occlusion can produce a rapid redistribution of blood volume from the occluded vascular territory towards higher compliance vascular compartment leading to changes in arterial system pressure and vascular resistance. Eventually, actual metaboreflex stimulation in term of autonomic adjustment was not directly assessed by microneurographic recordings of muscle sympathetic nerve activity (MSNA), limiting interpretation of post-exercise muscle ischemia hemodynamic response.

## 6 Conclusion

In conclusion, patients with CA-TTR display enhanced metaboreflex sensitivity, which is associated with sympathetic overdrive and exercise intolerance. We interpret our findings by postulating that a primary muscle disorder increases metaboreflex sensitivity, causing a chronic sympathovagal imbalance that can impair exercise aerobic capacity.

## Data Availability

The raw data supporting the conclusion of this article will be made available by the authors, without undue reservation.
